# Environmental Factors Correlated with Culturable Enterococci Concentrations in Tropical Recreational Waters: A Case Study in Escambron Beach, San Juan, Puerto Rico

**DOI:** 10.3390/ijerph14121602

**Published:** 2017-12-19

**Authors:** Abdiel E. Laureano-Rosario, Erin M. Symonds, Digna Rueda-Roa, Daniel Otis, Frank E. Muller-Karger

**Affiliations:** College of Marine Science, University of South Florida, Saint Petersburg, FL 33701, USA; esymonds@mail.usf.edu (E.M.S.); druedaro@mail.usf.edu (D.R.-R.); dotis@mail.usf.edu (D.O.); carib@usf.edu (F.E.M.-K.)

**Keywords:** recreational beach water quality, fecal indicator bacteria, coastal water quality, ocean color, remote sensing

## Abstract

Enterococci concentration variability at Escambron Beach, San Juan, Puerto Rico, was examined in the context of environmental conditions observed during 2005–2015. Satellite-derived sea surface temperature (SST), turbidity, direct normal irradiance, and dew point were combined with local precipitation, winds, and mean sea level (MSL) observations in a stepwise multiple regression analyses (Akaike Information Criteria model selection). Precipitation, MSL, irradiance, SST, and turbidity explained 20% of the variation in observed enterococci concentrations based upon these analyses. Changes in these parameters preceded increases in enterococci concentrations by 24 h up to 11 days, particularly during positive anomalies of turbidity, SST, and 480–960 mm of accumulated (4 days) precipitation, which relates to bacterial ecology. Weaker, yet still significant, increases in enterococci concentrations were also observed during positive dew point anomalies. Enterococci concentrations decreased with elevated irradiance and MSL anomalies. Unsafe enterococci concentrations per US EPA recreational water quality guidelines occurred when 4-day cumulative precipitation ranged 481–960 mm; irradiance < 667 W·m^−2^; daily average turbidity anomaly >0.005 sr^−1^; SST anomaly >0.8 °C; and 3-day average MSL anomaly <−18.8 cm. This case study shows that satellite-derived environmental data can be used to inform future water quality studies and protect human health.

## 1. Introduction

Fecal pollution is a threat to coastal ecosystems in many countries around the world that carries important public health and economic consequences. The city of San Juan, the capital of the island of Puerto Rico in the Caribbean Sea, is located within the Rio Piedras watershed, which receives the discharge of two centralized wastewater treatment plants (WWTPs; Puerto Nuevo Regional and Bayamon Regional WWTPs). The Rio Piedras watershed also catches the runoff from agricultural areas further upstream [1,2] as well as septic seepage [3,4,5]. While 56% of the Puerto Rican population is connected to these sewer systems [4,5], the remaining population, especially those located at higher elevations in San Juan, typically uses septic tanks. These septic tanks discharge approximately 165 million gallons per day directly to streams that empty into coastal waters [3,4,5]. Inadequate wastewater treatment prior to ocean outfall discharge, ineffective and old stormwater systems, and septic systems that leak into the karst geology and streams in the region are a constant and present danger to the public, especially because of the large numbers of people that enjoy visiting these beaches year-round for recreational purposes.

It is impractical to measure the concentrations of all wastewater-associated pathogens. Therefore, allochthonous gastrointestinal bacteria known as fecal indicator bacteria (FIB; e.g., fecal coliforms, *Escherichia coli*, and *Enterococcus* spp.), are used to characterize water quality [6,7]. While enterococci have correlated with public health risks in coastal areas with known point sources of fecal contamination in temperate and sub-tropical regions [7,8,9,10,11,12], this correlation has only been suggested in tropical regions [13] and has not been identified in areas exposed to non-point sources of fecal pollution [14,15]. Since FIB persist in the environment in the absence of active fecal pollution events, particularly in tropical climates, it is often difficult to differentiate between actual events that pose a threat to public health and the natural resuspension and growth of FIB in coastal waters [16,17]. Despite the environments important role on enterococci concentrations in tropical surface waters, few studies have investigated the relationship between environmental conditions and enterococci concentrations for beaches located in tropical climates [2,6,9,10,13].

The 2012 United States Environmental Protection Agency (US EPA) Recreational Water Quality Criteria (RWQC) recommends that culturable enterococci concentrations not exceed geometric means of 35 colony forming units (CFU) per 100 mL for safe recreation [18]. The Puerto Rico Environmental Quality Board (PREQB) has adopted this recommendation in their coastal recreational water quality monitoring program. Since October 2015, the public notification has been issued based on the Beach Action Value (BAV) of 70 CFU/100 mL, recommended by the US EPA National Beach Guidance and Required Performance Criteria for Grants [19,20]. The PREQB assesses beach water quality throughout the island every two weeks per the 2000 US Beaches Environmental Assessment and Coastal Health Act [14,21] and water quality standards of Puerto Rico [22].

Previous water quality studies in Puerto Rico have mostly focused on infrastructure; however, there have been a handful of short-term studies (i.e., weeks to months) on the relationship between water quality and environmental conditions [14,23,24,25]. These investigations showed that environmental parameters can influence the persistence and concentration of enterococci in recreational waters. For example, increased precipitation contributed to elevated enterococci concentrations [14]. Enterococci thrived in warmer waters [26] and were inhibited by increased irradiance [27]. Increased turbidity protected enterococci from ultraviolet (UV) light [28,29]. Enterococci concentrations in beach water also increased during low tide [30]. It is not known if enterococci co-occurred or correlated with the presence of wastewater-related pathogens or incidence of infection in these studies. Whether there is a relationship between FIB and long-term environmental change is a question that has not yet been explored.

The current case study seeks to identify environmental factors that influence the variability of culturable enterococci concentrations in Escambron Beach surface waters, and specifically seeks to assess when water quality issues exceed the US EPA recommended rate of 36 illnesses per 1000 primary contact recreators (BAV 70 CFU/100 mL; [18,20]). The approach included an analysis of 11 years of culturable enterococci concentrations with respect to the spatial and temporal variation of environmental factors observed locally (i.e., mean sea level, precipitation, winds) and via satellite (i.e., turbidity, sea surface temperature, dew point, direct normal irradiance). Previous studies in tropical areas have suggested a relationship between *Enterococcus* spp. and public health risks in recreational beach waters. However, the extra-intestinal sources of enterococci in the tropics as well as the presence of non-point sources of fecal pollution can obscure this relationship. Thus, it is important to understand how environmental factors influence culturable enterococci concentrations in tropical settings.

This case study demonstrates how the environmental factors significantly correlated with culturable enterococci surface water concentrations and considered the specific lags and ranges where these factors correlated with unsafe culturable enterococci concentrations at Escambron Beach. This study serves as an important point of reference for future water quality studies at Escambron beach as well as for other Caribbean beaches located in a similar context. The results of this case study begin to fill the existing knowledge gaps specific to water quality in the tropics and set the stage for targeted beach monitoring aimed at identifying a correlation between enterococci and health risks in the tropics. Additionally, the results can be used to develop microbial water quality forecasting systems, which would provide early information to local authorities, avoid unnecessary beach closures, and effectively balance the need to protect public health with the economic consequences associated with beach closures.

## 2. Materials and Methods

### 2.1. Escambron Beach, San Juan, Puerto Rico

**Figure 1:**
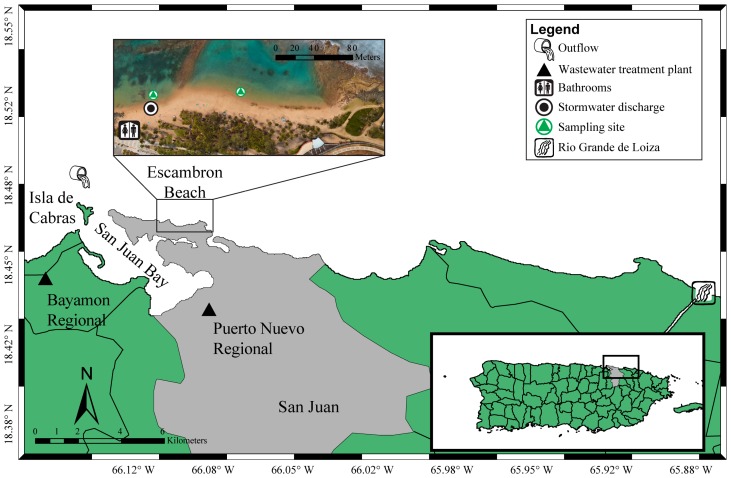
Location of Escambron Beach with respect to the combined ocean outfall that discharges primary-treated domestic wastewater from the Puerto Nuevo Regional and Bayamon Regional treatment plants (black triangles). The ocean outfall discharges at a depth of approximately 40 m; it is located 1 km north of Isla de Cabras and about 5 km from the study site. The inset map details Escambron Beach and depicts both sampling locations (green triangles), stormwater discharge drain (black circle), Rio Grande de Loiza (river symbol), and public bathrooms (bathroom symbol).

The study took place at Escambron Beach (18.47° N, 66.08° W, Figure 1). This is one of the most popular, well-visited beaches of San Juan, Puerto Rico and it has a year-long swimming season. Escambron Beach is generally visited by residents between the months of May to September, whereas during October to December it is mostly visited by non-residents and tourists. The beach is surrounded by hotels, business, residences, and governmental buildings [2]. The average annual air temperatures range between 24 and 29 °C. During the timeframe of this study, average precipitation was ~1800 mm per year, with lowest precipitation during February–March. Escambron Beach has mixed semidiurnal tides, and is classified as a low wave action along beach.

Two sites, separated by a distance of ~100 m, were sampled by the PREQB (Figure 1). These sites may have been affected by: (1) stormwater drainage (18.46° N, 66.09° W) located immediately adjacent to one of the sampling sites, which includes urban runoff, precipitation, and other graywaters (e.g., showers, washer machines; [2]); (2) WWTP ocean outfall (18.47° N, 66.14° W); (3) beach public bathrooms; and (4) Rio Grande de Loiza, a river that receives agricultural runoff, WWTP effluent (secondary treatment only), and septic system effluent and seepage (Figure 1; [31,32,33]). Non-point source pollution throughout San Jan Bay also likely affects water quality at Escambron Beach.

### 2.2. Culturable Enterococci Data

Data from biweekly measurements of culturable enterococci surface water concentrations were obtained for 2005–2012 from the US EPA Storage and Retrieval data warehouse for Escambron Beach (https://www.epa.gov/waterdata/water-quality-data-wqx). The enterococci time series was extended from 2012 to 2015 with data provided by PREQB. Two methods were used to quantify enterococci concentrations: US EPA method 1600 from January 2005–March 2015 [34] and IDEXX Enterolert (IDEXX Laboratories, Inc., Westbrook, ME, USA) from April 2015 through December 2015 [19]. The US EPA method 1600 quantifies culturable enterococci using membrane filtration and had a detection limit of 4 CFU/100 mL [34]. The IDEXX Enterolert Quanti-Tray methods determined the culturable enterococci concentrations (most probable number (MPN) per 100 mL); this method had a detection limit of 10 MPN/100 mL [19]. Over the 11-year period, a total of 642 culturable enterococci data points were generated, with the surface waters from two sites being sampled approximately once every two weeks (PREQB RW-20A at 18.47° N, 66.08° W and PREQB RW-20B at 18.46° N, 66.09° W; Figure 1). One site had 334 and the other site had 308 data points from 2005 to 2015. Daily geometric means for each sampling date, considering both sampling sites, were calculated for the beach; thus, the total combined number of unique enterococci data points was *n* = 376 for the 2005–2015 period. These geometric means were used in all further analyses. All samples were collected between 9:00 a.m. and 1:00 p.m. (AST). Puerto Rico’s climate does not show a significant season variability through the entire year. Therefore, samples collection considered sunlight amount, timing before people go to the beach, traveling time to the Puerto Rico Environmental Sciences Research Laboratory for sample processing, and timing of beach advisories if bacterial levels exceed thresholds [19,20]. If a sample had concentrations above US EPA guidelines, a subsequent sample was taken within seven days.

### 2.3. Satellite-Derived and In Situ Environmental Data

Daily precipitation data for San Juan, Puerto Rico were obtained from the US National Oceanic and Atmospheric Administration National Centers for Environmental Information for 2000–2015. Accumulated precipitation was calculated for different intervals of days prior to each surface water sampling date. Direct Normal Irradiance (DNI) and dew point were obtained at a 30-min temporal resolution and 4 km spatial resolutions from the National Solar Radiation Database (1998–2014; http://rredc.nrel.gov/solar/old_data/nsrdb/). Mean sea level (MSL) was obtained from the University of Hawaii Sea Level Center for 2000–2015 (https://uhslc.soest.hawaii.edu/). Maximum values, in a 24-h period, were identified for both MSL and DNI and included in the analyses due to their known influence on enterococci [29,30]. Wind speed and direction were obtained from the Caribbean Coastal Observing System (CariCOOS; http://www.caricoos.org/) buoy located north of San Juan (18.47° N, 66.09° W). East (u) and nort (v) wind components were calculated due to their mixing potential and influence on enterococci concentrations [35]. Since the CariCOOS buoy was deployed in 2010, satellite-derived wind data was also included from the Cross-Calibrated Multi-Platform (CCMP; ~28 km spatial resolution) surface winds (2010–2015). Both data sets were compared and followed the same patterns regarding wind speed, direction, and east/west components.

Sea surface temperature (SST) data were obtained from the Advanced Very High Resolution Radiometer (AVHRR; 1 km spatial resolution) from 2000 to 2015. These images were mapped using a cylindrical equidistant projection at the University of South Florida Institute for Marine Remote Sensing (http://imars.usf.edu/). Interactive Data Language (IDL; v. 7.2) was used to extract SST data from 3 × 3-pixel boxes centered on three points for the northern coast of San Juan, Puerto Rico (18.47° N, 66.09° W; 18.48° N, 66.08° W; 18.46° N, 66.07° W). Data from those three boxes showed similar temporal patterns; therefore, they were averaged into one SST time series for further analyses.

Remote sensing reflectance at 645 nm (R_rs_ 645) [36,37] from the Moderate Resolution Imaging Spectroradiometer (MODIS-Terra; 250 m spatial resolution) was used as a proxy for water turbidity (2005–2015). Remote sensing reflectance represents the ratio of upwelling “water-leaving” radiance to downwelling irradiance measured in per steradian (sr^−1^) units. Reflectance in the red (645 nm) is used here as a proxy for turbidity, an approach which has been used in several previous studies [36,38,39]. R_rs_ was extracted using MATLAB (v. 2014b; The MathWorks Inc., Natick, MA, USA, 2000); the average value of two 3 × 3-pixel boxes was used for turbidity for this coastal region (centered on: 18.47° N, 66.10° W; 18.46° N, 66.08° W; these included sampling sites and adjacent areas). Turbidity measurements from these boxes followed similar temporal patterns; thus, the data were averaged into a single time series for further analyses. Daily and weekly time series, climatologies, and anomalies were calculated for all the variables mentioned above for the period of 2005–2015. Both SST and turbidity, as extracted, covered the entire study site; however, for SST we added a third sampling point to cover the overall variability due to its lower spatial resolution (1 km) in comparison to turbidity (250 m). Additionally, 3-day averages of SST and turbidity anomaly images were also computed for the coastal region of the municipality of San Juan (l8.51–18.42° N, 66.16–65.85° W) to examine the spatial distribution of SST and turbidity before beach advisories on 9 March 2007 and 16 December 2011 (dates were selected based on satellite images availability/clarity to show data).

### 2.4. Data Identified as Below the Limit of Detection

Sixty-two enterococci data points out of the 376 (combined sampling sites) were described as below the limit of detection (LOD); consequently, it was necessary to accommodate these data to be able to use the 2005–2015 data set for downstream analyses. To determine the most appropriate substitution [40,41,42], the use of three different, previously-used substitutions were evaluated: the maximum concentration after the LOD (i.e., 3 CFU/100 mL and 9 CFU/100 mL for method 1600 and IDEXX Enterolert, respectively), minimum concentration (i.e., 1 CFU/100 mL), and half the detection limit (i.e., 2 CFU/100 mL and 5 CFU/100 mL). When comparing the three methods, all the correlations coefficients showed a difference less than 0.10 and were considered not significantly different. Based on this, it was concluded that the results of the downstream Akaike Information Criteria analyses were not significantly different among the three aforementioned substitution approaches (Supplementary Materials, Tables S1–S3). Therefore, a conservative approach was selected, such that all that left-censored data were substituted by the next highest concentration; 3 CFU per 100 mL for those samples analyzed before April 2015 and 9 MPN per 100 mL for samples analyzed after April 2015. These were substituted in the raw data (1999–2015) and then filtered to obtain a total of 376 points from 2005 to 2015.

### 2.5. Non-Parametric Statistical Analyses

Data were analyzed with non-parametric, permutation-based statistics, which are a distribution-free method. Significant time-lagged correlations between explanatory variables (i.e., SST, precipitation, DNI, dew point, MSL, and turbidity) and the dependent variable (i.e., culturable enterococci concentrations) were identified using Pearson’s correlation analyses. A MATLAB function was created to identify different lags between explanatory variables and dependent variable, where those who showed the highest and significant Person’s correlation coefficient were selected.

Lagged environmental factors, with the lag-periods showing significant correlations (Pearson’s correlations) with culturable enterococci were divided into three to six bins using the histogram function of MATLAB (v.2014b); bins sizes were selected based upon sample size. Bins were divided as follow: (A) precipitation (mm) six bins: ≤240, 241–480, 481–720, 721–960, and ≥961; (B) DNI (W·m^−2^) five bins: ≤667, 668–732, 733–798, 799–864, and ≥865; (C) turbidity anomaly (sr^−1^) three bins: ≤0.001, 0.002–0.004, and ≥0.005; (D) SST anomaly (°C) four bins: ≤−3.7, −3.6–−1.5, −1.4–0.7, and ≥0.8; (E) dew point anomaly (°C) five bins: ≤−1.6, −1.5–−0.7, −0.6–−0.3, −0.2–1.2, and ≥1.3; and (F) MSL anomaly (cm) six bins: ≤−78.8, −78.7–−18.8, −18.7–41.2, 41.3–101.2, 101.3–161.2, and ≥161.3. Surface water sampling dates that matched those specific ranges in environmental conditions were identified and the average geometric means (of enterococci concentrations) were extracted for each bin. Confidence intervals for each of the bins were calculated using bootstrapping (random sampling with replacement) with 5000 iterations. Subsequently, permutation-based one-way ANOVAs were executed for each explanatory variable to test for significance across different environmental parameter ranges. For those intervals that showed a significant or marginally non-significant difference, a series of a posteriori, multiple-comparison (pair-wise) tests were run to identify those bins that were significantly different.

A stepwise selection of explanatory variables via forward addition based on Akaike Information Criteria (AIC) was executed. AIC analyses identified the optimal environmental factors that substantially explained variation in culturable enterococci concentrations [43,44,45]. The variables included in the AIC analysis were precipitation, SST, dew point, MSL, DNI, and turbidity. The MATLAB Fathom toolbox was used for all data analyses [46].

## 3. Results

### 3.1. Modeling Culturable Enterococci Using Akaike Information Criterion Model and Correlation Analyses

The environmental variables used in the AIC model were selected based on their significant, time-lagged correlations identified by the Pearson’s correlation analyses (*p* < 0.05; Table 1). The stepwise AIC analyses showed that precipitation, MSL, DNI, SST, and turbidity were the optimal explanatory variables for culturable enterococci concentrations in Escambron Beach surface waters during 2005–2015 (*p* < 0.05; *r*^2^ = 0.20; Table 2); dew point was not identified as an optimal explanatory variable by the AIC analyses. 

### 3.2. Environmental Variables Influence on Culturable Enterococci

**Figure 2:**
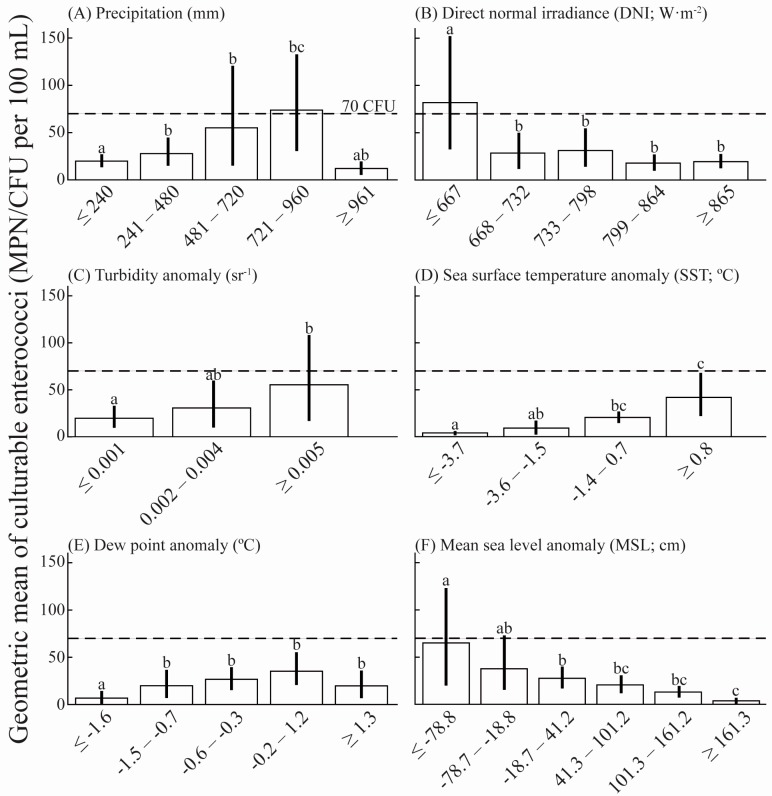
Geometric mean of enterococci concentrations in Escambron Beach surface waters at different ranges of (**A**) precipitation; (**B**) direct normal irradiance (DNI); (**C**) turbidity anomaly; (**D**) sea surface temperature anomaly (SST); (**E**) dew point anomaly; and (**F**) mean sea level (MSL) anomaly at Escambron Beach during 2005–2015. Dashed lines are the 2014 US EPA beach action value (BAV) of 70 CFU/100 mL (US EPA 2012; 2014). Vertical lines represent the 95% confidence interval. Lower-case letters above the vertical lines identify statistically significant differences among bins (α = 0.05).

All the variables were divided into three to six bins to characterize how culturable enterococci concentrations were influenced across specific ranges of environmental variables (Figure 2A–F). Precipitation, SST, dew point, and turbidity anomalies showed a positive correlation with enterococci concentrations in Escambron Beach surface waters (Table 1). Enterococci concentrations were above the 2014 US EPA BAV and exceeded the 2012 US EPA RWQC recommendation 1 (36 estimated illnesses per 1000 recreators, [18,20]) after 481 mm–960 mm of rainfall in four days, SST greater than 0.8 °C for at least 5 days, or turbidity anomalies greater than 0.005 sr^−1^ after 24 h (Figure 2). During warmer anomalies of high dew points sustained over seven consecutive days, there were higher concentrations of culturable enterococci. (Table 1). While these conditions did not automatically lead to levels exceeding the 2014 US EPA BAV, they did lead to values above the original 2012 US EPA RWQC recommendation 1 (Figure 2E).

Conversely, DNI and MSL showed a strong, negative correlation with enterococci concentrations in Escambron Beach surface waters. The highest correlations were observed after a 24-h lag for DNI, and a nine-day lag for MSL anomalies (Table 1). Higher culturable enterococci concentrations, exceeding the 2014 US EPA BAV and 2012 US EPA RWQC recommendation 1, were observed during the lowest DNI (≤667 W·m^−2^; Figure 2B) as well as during the lowest negative MSL anomalies (≤−18.8 cm; Figure 2F). Culturable enterococci concentrations decreased as DNI increased (>~668 W·m^−2^; Figure 2B). Wind data (i.e., average wind speed, u-component, and v-component) showed no significant correlation with culturable enterococci during 2005–2015 (data not shown).

### 3.3. Satellite-Derived SST and Turbidity Anomaly Images to Anticipate Potential Beach Advisories

**Figure 3:**
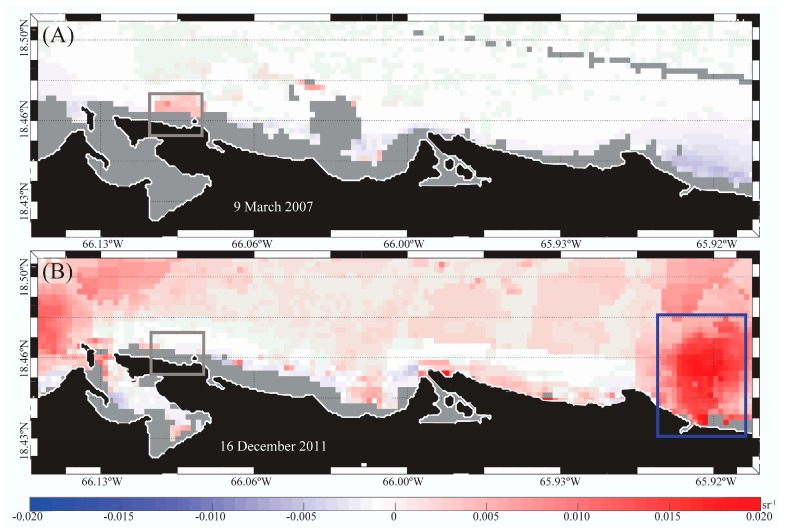
Anomalies of remote sensing reflectance (R_rs_ 645 nm, 250 m spatial resolution, from the Moderate Resolution Imaging Spectroradiometer MODIS-Terra) showing Escambron Beach (gray box) water clarity anomalies three days before the beach advisories of (**A**) 9 March 2007 and (**B**) 16 December 2011. Blue box on (**B**) shows the high discharge of Rio Grande de Loiza on 16 December 2011.

**Figure 4:**
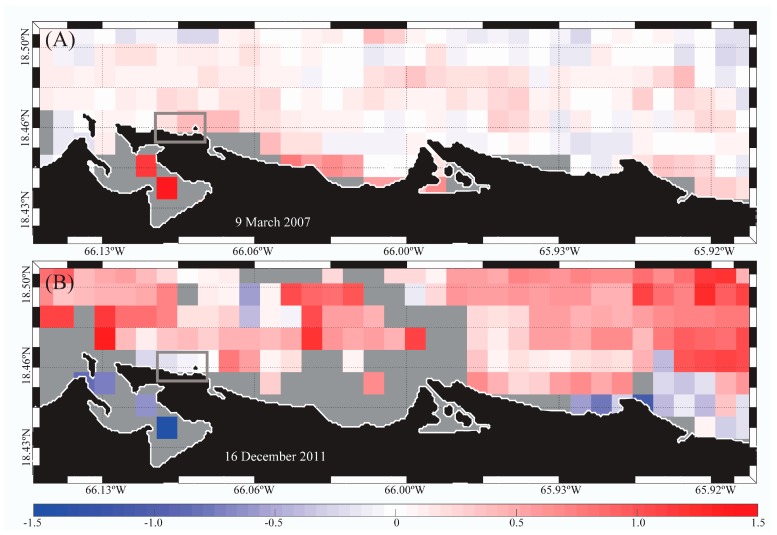
Sea Surface Temperature (SST) anomalies (from the Advanced Very High Resolution Radiometer-AVHRR; 1km spatial resolution) showing Escambron Beach (gray box) three days before the beach advisories of (**A**) 9 March 2007; and (**B**) 16 December 2011.

The PREQB issued beach advisories on 9 March 2007 (>35 enterococci CFU/100 mL) and 16 December 2011 (48 enterococci CFU/100 mL), where the possible sources were identified as sewer line and runoff, respectively (US EPA 2016). The mean satellite-derived turbidity over three consecutive days prior to these events was higher than normal in adjacent areas (Figure 3; gray boxes). Higher than normal river discharge (i.e., >~0.2 m^3^·s^−1^ based on anomalies from 1998 to 2015; data not shown) was observed on 16 December 2011 (Figure 3B; Rio Grande de Loiza; dark blue box). Warmer than normal waters were also observed on both dates (Figure 4) over the whole region. SST on 16 December 2011 was even warmer (~0.5–1.0 °C) than on 9 March 2007 (Figure 4).

## 4. Discussion

In US recreational waters, enterococci continue to be the recommended fecal pollution indicator despite their natural presence in tropical waters [6,7,47,48]. Thus, it is necessary to understand the environmental factors related to elevated FIB concentrations to begin to differentiate pollution events from FIB ecology as well as to forecast water quality. The current case study investigated the environmental conditions that are related to exceeding the recommended enterococci concentrations at a Caribbean beach. While similar studies have been executed in other tropical [13,49] and subtropical locations [50,51,52,53] using in situ environmental data, this study also incorporated satellite-derived environmental data. In addition to identifying the time-lagged correlations of environmental parameters and culturable enterococci concentrations, the environmental parameter ranges and patterns were also identified when enterococci concentrations indicated unsafe recreational water quality (>70 CFU/100 mL; 2014 US EPA BAV). While future research is needed to identify if enterococci correlate with human health risks, this study provides an overview of how long-term data can be used to understand the most influential environmental variables on culturable enterococci concentrations and sets the stage for future investigations to distinguish the cause of high enterococci concentrations (fecal pollution events vs. enterococci ecology).

### 4.1. Environmental Factors Associated with Culturable Enterococci Variability

Of the environmental factors analyzed in this study, only precipitation, DNI, MSL, SST, and turbidity were significantly associated with enterococci concentrations; these associations may have been due to enterococci ecology and/or fecal pollution events. The strong correlation between precipitation, particularly 4-day accumulated rain events, and enterococci concentrations may indeed be explained by increased sewage and septic tank discharge [2,14,54,55,56], or runoff with animal feces [9,25,57,58]. However, it is also possible that precipitation increased the presence of non-fecal sources of enterococci via resuspension of sediments as well as runoff of bacteria in soil [35,36]. With respect to lower enterococci concentrations outside the 481 mm–960 mm precipitation range, it is possible that drier conditions promoted decreased bacterial replication due to lower nutrient additions and/or reduced stormwater inputs decreased the input of enterococci into beach surface waters. With respect to the wettest conditions, it is possible that the excess rainfall diluted enterococci concentrations in surface waters [59,60].

Similarly, the significant decrease observed in enterococci concentrations during periods of high solar irradiance was likely due to production of reactive oxygen species (ROS) that cause bacterial dieoff [27,30,61]; however, enterococci concentrations may have been low due to a lack of fecal pollution inputs (e.g., stormwater, runoff). There was also an inverse correlation between enterococci concentrations and MSL anomalies. It is possible that bacterial dilution occurs during higher MSL anomalies, and concomitant back-washing mixing and enhanced drainage from coastal sources may promote increased bacteria concentrations during lower MSL anomalies [30,62,63]. While winds have been previously correlated with increased enterococci concentrations [35,64], no correlation between wind components and culturable enterococci concentrations was identified for Escambron Beach surface waters during 2005–2015. It is possible that the limited local wind data obscured the identification of significant correlations between winds and enterococci concentrations. Additionally, increased enterococci concentrations were observed during warmer SST anomalies, which could be due to an increased bacterial growth and replication (e.g., metabolism) due to warmer water temperatures [35,48]. Finally, this study did not consider how the presence of aquatic plants, such as seagrass, or algae, such as the green alga *Cladophora*, could have decreased and increased enterococci concentrations, respectively [65,66,67,68].

Given the combined effects between many of the environmental parameters analyzed, it is difficult to tease apart their independent influence on coastal enterococci concentrations. For example, higher turbidity anomalies are the result of increased runoff [69,70], but increased enterococci concentrations could also be the result of protection from UV exposure [28,29,71]. Furthermore, it is possible that dew point was not identified as an optimal explanatory variable due to its relationship with rainfall as well as to SST [72] as the AIC model reduces multi-collinearity (i.e., higher correlations between predictor variables). Finally, winds are known to be associated with increased precipitation, which also leads to increased wave action that stimulate sediment resuspension, which can increase non-fecal enterococci concentrations [35,58,64]. While it is difficult to discern the confounding influence of each environmental parameter, this study identified the environmental conditions that should be considered for microbial water quality modeling at Escambron Beach.

Even though this study was not able to tease apart the combined effects of environmental variables nor enterococci ecology from actual fecal pollution events, the results of this investigation demonstrated that precipitation, DNI, MSL, SST, and turbidity were strongly and significantly associated with culturable enterococci concentrations at a tropical, Caribbean beach. Similar results have been shown in freshwaters, where enterococci concentrations were modeled in the great lakes and parameters, such as river discharge, temperature, turbidity, and winds were significantly correlated with enterococci variability [73,74]. Liu et al. [75] showed that human fecal pollution is also transported in river tributaries, where discharges into the great lakes influence enterococci concentrations and create public health concerns. Now that the environmental conditions associated with enterococci concentrations exceeding the limits for safe recreation have been identified for Escambron Beach, future research is needed to tease apart the influence of enterococci ecology versus fecal pollution events and should include microbial source tracking and measurement of human pathogens.

### 4.2. Sanitation Infrastructure, Human Fecal Pollution, and Culturable Enterococci Variability

Since only 20% of the enterococci variation was explained by the environmental parameters in the AIC model in this study, it is possible that the remaining 80% could have been attributed to sanitation infrastructure (which was not included in the analyses), other environmental factors (e.g., extra-intestinal, environmental enterococci sources; animal feces), as well as stochastic variability [35]. About 42% of the people living in Puerto Rico use septic tanks and many of these systems do not work properly or lack maintenance [4]. Consequently, improperly functioning systems and the porous karst-geology facilitate the movement of domestic wastewater into surrounding surface waters [1,4]. Over the last 50 to 60 years, there has been a shift from septic tanks to centralized WWTP (primary treatment) to accommodate San Juan’s growing population and increasing urbanization. Escambron Beach also has public bathrooms located next to its stormwater discharge. While toilets are connected to the centralized sewer system, there may be leaks that can influence enterococci concentrations at the study site [2].

The combined Puerto Nuevo Regional and Bayamon Regional WWTP ocean outfall is located approximately 5 km northwest from Escambron Beach. Yet this discharge can impact beach water quality under specific current regimes. The outfall discharges ~200 MGD of primary-treated domestic wastewater at 40 m depth [76], which vertically mixes due to buoyancy forces and causes the development of an ocean outfall surface and sub-surface boils [77]. Following initial mixing, ocean currents can transport and dilute the outfall boil. Currents in Puerto Rico are generally westward and influenced by the westerlies; however, the CariCOOS buoy current data shows very weak south-southeast semi-diurnal tidal currents on Puerto Rico’s northern coast between 2 and 30 m depth. Any episode that strengthens this eastward flow can carry outfall boils toward Escambron Beach. Additionally, the Rio Grande de Loiza discharge is another potential source of contamination due to westward movement of currents. Studies have shown that this river’s tributaries were impaired due to fecal contamination [32,33,78]; thus, the Rio Grande de Loiza discharge could have impacted the study area, particularly when flow rates were high during rain events [79].

### 4.3. Satellite-Derived SST and Turbidity Anomaly Images, and PREQB Beach Advisories

Beach advisories are issued by PREQB two to three days after the sampling date when culturable enterococci concentrations exceeded the PREQB water quality criteria. Consequently, it is important to understand the lags, ranges, and spatial distributions of precipitation, DNI, MSL, SST, and turbidity, that are correlated with culturable enterococci concentrations to identify conditions that lead to such advisories [80]. Additionally, it is well-known that enterococci may not be the most appropriate water quality indicator for tropical regions due to its presence in secondary, non-fecal related reservoirs that confound the identification of health risks (e.g., soil, sediments) [17,49]. Thus, this understanding can inform future studies that seek to decipher when enterococci might exceed US EPA guidelines and represent an actual health risk versus when no health risk is present [17].

For example, high turbidity and SST anomalies occurred at Escambron beach during the days preceding the 9 March 2007 and 16 December 2011 beach closures. Prior to the 16 December 2011 advisory, there was a significant discharge from the Rio Grande de Loiza, which was likely transported toward the beach by westward ocean currents [1] and likely caused the high turbidity anomalies observed. While there was no anomalous river discharge during 9 March 2007, higher than normal turbidity was observed north of the study area. Additionally, the images showed warmer than normal water temperatures (~1.0–1.5 °C) for most of the region, which also likely influenced increased culturable enterococci concentrations. Since these satellite-derived data preceded the beach closures, satellite-derived data can help identify conditions for poor water quality in advance and guide sampling efforts.

### 4.4. Future Work

A better understanding of enterococci variability with respect to environmental conditions and fecal pollution events is needed to identify accurately public health risks and minimize public exposure to such risks [10,81,82]. Ideally, these risks should be forecasted by beach-specific predictive models to prevent the public’s exposure to waterborne pathogens [28,83]. To create such models, future investigations at Escambron Beach should consider this study’s results, which identified that precipitation, DNI, MSL, SST, and turbidity significantly influence enterococci concentrations, as well as the following: (1) the relationship between enterococci and human health risks (e.g., illness, reference pathogens) and (2) the impact of human (e.g., WWTP ocean outfall, leaky septic systems) and non-human (e.g., animal feces) fecal pollution sources that can influence enterococci variability and/or health risks. Additionally, data were not analyzed in terms of wet/dry season because the dry season at Escambron beach is only two-months long (February and March) and therefore, requires a different data set to achieve sufficient statistical power to determine how environmental variability influences enterococci concentrations by season.

Since the Puerto Rican economy relies mostly on tourism, proper management of Escambron Beach through targeted monitoring and early-warning systems is necessary to restrict beach advisories/closures to those that are truly necessary to protect public health [51,78]. This investigation demonstrated that satellite-derived and local environmental parameters explained enterococci variability at a tropical, Caribbean beach. The results presented will be important to future water quality investigations in the tropics, as well as to the development of the spatial and temporal components of predictive models that aim to improve forecasting and now-casting of beach water quality.

## 5. Conclusions

Identifying the environmental factors correlated with culturable enterococci concentrations can help to better understand their potential risk to public health in a tropical setting. This study looked into 11 years of culturable enterococci concentrations and assessed how much of its variability can be explained by environmental factors alone, where the main findings were:

- Environmental factors (i.e., direct normal irradiance (DNI), mean sea level (MSL), precipitation, turbidity, and sea surface temperature (SST)) explained 20% of the enterococci variability observed in Escambron Beach surface waters during 2005–2015.
- Identified time-lags for the different environmental factors helped better understand variability in culturable enterococci in Escambron Beach surface waters due to environmental factors.
- Increased enterococci concentrations were observed at strong positive SST and turbidity anomalies; conversely, these concentrations decreased with increased DNI and MSL anomalies in Escambron Beach surface waters.
- Specific ranges of precipitation (i.e., 481–960 mm) promoted increased enterococci concentrations, potentially due to urban run-off (e.g., resuspension of solids, soil runoff, and non-human sources), combined sewer overflow events, and/or increased leaching of septic tanks.
- The combined effects of environmental factors can help model culturable enterococci concentrations and understand ranges where these would exceed recommended 2014 US EPA BAV at Escambron Beach.
- Satellite-derived data can improve beach water quality assessments, potentially reducing in-situ sampling efforts as this data is readily available, and help identify events for early warning systems and improve beach advisory.

## Figures and Tables

**Table 1:** Pearson’s correlation coefficient to identify significant lags in enterococci concentrations in surface waters at Escambron Beach with respect to the environmental parameters: Mean sea level (MSL), direct normal irradiance (DNI), sea surface temperature (SST), dew point, turbidity, and precipitation. Values are considered significant at α < 0.05.

Variable	Pearson’s Correlation Coefficient (*r*)	Lag
Mean sea level	−0.19	9th to 11th day (mean)
Direct normal irradiance	−0.24	1 day
Sea surface temperature	0.12	5th to 9th day (mean)
Dew point	0.19	7 days (mean)
Turbidity	0.25	1 day
Precipitation	0.22	4 days (accumulated)

**Table 2:** Akaike Information Criterion (AIC) model with those environmental variables that explained enterococci concentration variability in surface waters at Escambron Beach.

Variable	*r*^2^	*r*^2^-Adjusted	AIC
Precipitation	0.08	0.08	59.81
Mean sea level	0.13	0.12	48.76
Direct normal irradiance	0.16	0.15	39.19
Sea surface temperature	0.19	0.17	32.79
Turbidity	0.21	0.19	26.76

## Supplementary Materials

The following are available online at www.mdpi.com/1660-4601/14/12/1602/s1, Table S1: Pearson’s correlation coefficient to identify significant lags in enterococci concentrations in Escambron Beach surface waters with respect to the environmental parameters. Concentrations below the limit of detection were substituted by 1 MPN/CFU per 100 mL. Values are considered significant with 95% certainty (α = 0.05). Bold values are those with the highest and significant Pearson’s correlation coefficient, Table S2: Pearson’s correlation coefficient to identify significant lags of enterococci concentrations in Escambron Beach surface waters with respect to the environmental parameters. Concentrations below the limit of detection were substituted by 3 MPN/CFU per 100 mL for those samples analyzed before April 2015 and 9 MPN/100 mL for samples analyzed after April 2015. Values are considered significant with 95% certainty (α = 0.05). Bold values are those with the highest and significant Pearson’s correlation coefficient, Table S3: Pearson’s correlation coefficient to identify significant lags of enterococci concentrations in Escambron Beach surface waters with respect to the environmental parameters. Concentrations below the limit of detection were substituted by 2 MPN/CFU per 100 mL for those samples analyzed before April 2015 and 5 MPN/100mL for samples analyzed after April 2015. Values are considered significant with 95% certainty (α = 0.05). Bold values are those with the highest and significant Pearson’s correlation coefficient.
