# Supplementary material for: Environmental Factors Correlated with Culturable Enterococci Concentrations in Tropical Recreational Waters: A Case Study in Escambron Beach, San Juan, Puerto Rico
Source: Int J Environ Res Public Health. 2017 Dec 19;14(12):1602. doi: 10.3390/ijerph14121602 (PMC5751019; doi:10.3390/ijerph14121602)
Supplement: Supplementary file 1 [file ijerph-14-01602-s001.pdf]

International Journal of
Environmental Research and Public Health

MDPI

Supplementary Materials: Environmental Factors Correlated with Culturable Enterococci Concentrations in Tropical Recreational Waters: A Case Study in Escambron Beach, San Juan, Puerto Rico

Abdiel E. Laureano-Rosario *, Erin M. Symonds, Digna Rueda-Roa, Daniel Otis and Frank E. Muller-Karger

Table S1. Pearson's correlation coefficient to identify significant lags in enterococci concentrations in Escambron Beach surface waters with respect to the environmental parameters. Concentrations below the limit of detection were substituted by 1 MPN/CFU per 100 mL. Values are considered significant with 95% certainty (α = 0.05). Bold values are those with the highest and significant Pearson's correlation coefficient.

Precipitation			Sea Surface Temperature			Dew Point			Mean Sea Level			Direct Normal Irradiance			Turbidity		
Lag	r	*p*-value	Lag	r	*p*-value	Lag	r	*p*-value	Lag	r	*p*-value	Lag	r	*p*-value	Lag	r	*p*-value
1	0.12	0.0278	1	0.05	0.3504	1	0.15	0.0086	1	0.09	0.1064	1	−0.25	0.0004	1	0.23	0.0235
2	0.23	0.0002	2	0.07	0.2150	2	0.17	0.0042	2	0.07	0.2376	2	−0.22	0.0010	2	0.05	0.6267
**3**	**0.23**	**0.0002**	3	0.08	0.1604	3	0.17	0.0028	3	0.05	0.3720	3	−0.22	0.0006	3	−0.02	0.8455
**4**	**0.23**	**0.0004**	4	0.08	0.1688	4	0.19	0.0028	4	0.01	0.8484	4	−0.22	0.0002	4	−0.17	0.1049
5	0.22	0.0002	**5**	**0.12**	**0.0436**	5	0.20	0.0008	5	−0.04	0.4962	5	−0.22	0.0002	5	0.13	0.2510
6	0.19	0.0010	6	0.07	0.2382	6	0.20	0.0004	6	−0.11	0.0502	6	−0.20	0.0004	6	0.03	0.7851
7	0.18	0.0014	7	0.10	0.0790	**7**	**0.21**	**0.0006**	7	−0.17	0.0028	7	−0.20	0.0014	7	0.08	0.4977
8	0.17	0.0050	8	0.08	0.1796	8	0.20	0.0006	8	−0.21	0.0006	8	−0.19	0.0014	8	−0.15	0.1640
9	0.15	0.0108	9	0.06	0.2708	9	0.20	0.0010	**9**	**−0.23**	**0.0004**	9	−0.17	0.0040	9	0.11	0.3536
10	0.14	0.0154	10	0.01	0.8526	10	0.20	0.0010	10	−0.22	0.0002	10	−0.17	0.0042	10	0.00	0.9726
11	0.14	0.0140	11	0.04	0.4992	11	0.20	0.0004	11	−0.19	0.0012	11	−0.17	0.0062	11	0.16	0.1341
12	0.14	0.0158	12	0.00	0.9968	12	0.20	0.0006	12	−0.13	0.0214	12	−0.16	0.0060	12	−0.15	0.1564
13	0.14	0.0130	13	−0.03	0.6000	13	0.20	0.0008	13	−0.05	0.3908	13	−0.16	0.0068	13	−0.12	0.2779
14	0.12	0.0350	14	−0.03	0.6330	14	0.20	0.0018	14	0.00	0.9984	14	−0.15	0.0126	14	−0.15	0.1561
15	0.12	0.0376	15	−0.03	0.6562	15	0.20	0.0008	15	0.04	0.4748	15	−0.14	0.0182	15	−0.05	0.6400
16	0.11	0.0450	16	0.01	0.9226	16	0.19	0.0016	16	0.04	0.5312	16	−0.14	0.0220	16	−0.01	0.9330
17	0.12	0.0380	17	−0.02	0.7208	17	0.18	0.0026	17	0.02	0.7450	17	−0.14	0.0250	17	−0.15	0.1591
18	0.13	0.0238	18	−0.03	0.5506	18	0.18	0.0028	18	−0.03	0.6560	18	−0.13	0.0290	18	−0.08	0.5045
19	0.13	0.0188	19	0.00	0.9512	19	0.18	0.0028	19	−0.07	0.2368	19	−0.14	0.0230	19	0.07	0.5134
20	0.14	0.0178	20	0.03	0.5778	20	0.18	0.0030	20	−0.10	0.0824	20	−0.14	0.0272	20	−0.03	0.8150
21	0.12	0.0310	21	0.05	0.3852	21	0.18	0.0020	21	−0.14	0.0200	21	−0.13	0.0296	21	0.00	0.9687

Table S2. Pearson's correlation coefficient to identify significant lags of enterococci concentrations in Escambron Beach surface waters with respect to the environmental parameters. Concentrations below the limit of detection were substituted by 3 MPN/CFU per 100 mL for those samples analyzed before April 2015 and 9 MPN/ 100mL for samples analyzed after April 2015. Values are considered significant with 95% certainty (α = 0.05). Bold values are those with the highest and significant Pearson's correlation coefficient.

**Precipitation**			**Sea Surface Temperature**			**Dew Point**			**Mean Sea Level**			**Direct Normal Irradiance**			**Turbidity**		
Lag	r	*p*-value	Lag	r	*p*-value	Lag	r	*p*-value	Lag	r	*p*-value	Lag	r	*p*-value	Lag	r	*p*-value
1	0.13	0.0234	1	0.07	0.2196	1	0.13	0.0234	1	0.11	0.0594	1	−0.24	0.0002	1	0.25	0.0150
2	0.23	0.0004	2	0.09	0.1252	2	0.15	0.0102	2	0.08	0.1694	2	−0.21	0.0006	2	0.04	0.7100
**3**	**0.23**	**0.0002**	3	0.09	0.0962	3	0.15	0.0102	3	0.06	0.2728	3	−0.20	0.0010	3	−0.03	0.7696
**4**	**0.22**	**0.0006**	4	0.10	0.0930	4	0.17	0.0028	4	0.02	0.7198	4	−0.21	0.0012	4	−0.19	0.0700
5	0.21	0.0004	**5**	**0.12**	**0.0428**	5	0.18	0.0018	5	−0.03	0.6370	5	−0.20	0.0008	5	0.11	0.2838
6	0.17	0.0026	6	0.08	0.1796	6	0.18	0.0022	6	−0.10	0.0812	6	−0.18	0.0030	6	−0.01	0.9500
7	0.15	0.0088	7	0.11	0.0672	**7**	**0.19**	**0.0014**	7	−0.15	0.0066	7	−0.17	0.0038	7	0.09	0.4264
8	0.15	0.0072	8	0.08	0.1782	8	0.19	0.0018	8	−0.19	0.0016	8	−0.17	0.0046	8	−0.17	0.1161
9	0.13	0.0184	9	0.06	0.3234	9	0.18	0.0022	**9**	**−0.19**	**0.0012**	9	−0.15	0.0126	9	0.10	0.3817
10	0.12	0.0290	10	0.02	0.7010	10	0.18	0.0024	10	−0.18	0.0030	10	−0.15	0.0132	10	0.00	0.9630
11	0.12	0.0356	11	0.06	0.3414	11	0.19	0.0020	11	−0.13	0.0226	11	−0.15	0.0146	11	0.15	0.1362
12	0.12	0.0488	12	0.02	0.6708	12	0.19	0.0012	12	−0.08	0.1874	12	−0.14	0.0168	12	−0.16	0.1264
13	0.12	0.0444	13	−0.01	0.8966	13	0.18	0.0018	13	0.00	0.9530	13	−0.14	0.0176	13	−0.11	0.3476
14	0.10	0.0832	14	−0.01	0.8910	14	0.18	0.0018	14	0.04	0.4280	14	−0.13	0.0308	14	−0.14	0.1859
15	0.10	0.0956	15	−0.01	0.9104	15	0.18	0.0014	15	0.08	0.1590	15	−0.12	0.0392	15	−0.05	0.6391
16	0.09	0.1126	16	0.03	0.6328	16	0.17	0.0038	16	0.07	0.2022	16	−0.12	0.0506	16	−0.01	0.9101
17	0.09	0.1040	17	0.00	0.9874	17	0.17	0.0046	17	0.05	0.3648	17	−0.12	0.0480	17	−0.17	0.1077
18	0.10	0.0724	18	−0.01	0.8112	18	0.16	0.0064	18	0.01	0.9166	18	−0.11	0.0540	18	−0.06	0.6013
19	0.10	0.0694	19	0.02	0.6892	19	0.16	0.0068	19	−0.04	0.5060	19	−0.12	0.0522	19	0.09	0.4156
20	0.10	0.0612	20	0.06	0.3450	20	0.16	0.0064	20	−0.06	0.2552	20	−0.12	0.0486	20	−0.06	0.5993
21	0.09	0.1056	21	0.09	0.1220	21	0.17	0.0044	21	−0.10	0.0886	21	−0.11	0.0588	21	−0.01	0.9104

Table S3. Pearson's correlation coefficient to identify significant lags of enterococci concentrations in Escambron Beach surface waters with respect to the environmental parameters. Concentrations below the limit of detection were substituted by 2 MPN/CFU per 100 mL for those samples analyzed before April 2015 and 5 MPN/ 100mL for samples analyzed after April 2015. Values are considered significant with 95% certainty (α = 0.05). Bold values are those with the highest and significant Pearson's correlation coefficient.

**Precipitation**			**Sea Surface Temperature**			**Dew Point**			**Mean Sea Level**			**Direct Normal Irradiance**			**Turbidity**		
Lag	r	*p*-value	Lag	r	*p*-value	Lag	r	*p*-value	Lag	r	*p*-value	Lag	r	*p*-value	Lag	r	*p*-value
1	0.13	0.0184	1	0.07	0.2504	1	0.15	0.0130	1	0.10	0.0714	1	−0.25	0.0002	1	0.25	0.0153
2	0.23	0.0002	2	0.09	0.1452	2	0.16	0.0070	2	0.07	0.1988	2	−0.21	0.0008	2	0.05	0.6674
**3**	**0.23**	**0.0002**	3	0.09	0.1226	3	0.16	0.0072	3	0.06	0.3266	3	−0.21	0.0006	3	−0.02	0.8347
**4**	**0.23**	**0.0006**	4	0.09	0.1046	4	0.18	0.0032	4	0.01	0.8076	4	−0.21	0.0010	4	−0.19	0.0827
5	0.21	0.0004	**5**	**0.12**	**0.0408**	5	0.20	0.0010	5	−0.04	0.5224	5	−0.21	0.0006	5	0.12	0.2557
6	0.18	0.0026	6	0.08	0.1896	6	0.20	0.0008	6	−0.11	0.0554	6	−0.19	0.0020	6	0.00	0.9898
7	0.16	0.0032	7	0.11	0.0618	**7**	**0.20**	**0.0012**	7	−0.16	0.0038	7	−0.18	0.0034	7	0.09	0.4446
8	0.16	0.0086	8	0.08	0.1856	8	0.20	0.0012	8	−0.20	0.0008	8	−0.17	0.0034	8	−0.16	0.1262
9	0.14	0.0138	9	0.06	0.2966	9	0.19	0.0014	**9**	**−0.21**	**0.0002**	9	−0.16	0.0100	9	0.10	0.3723
10	0.13	0.0218	10	0.02	0.7396	10	0.19	0.0014	10	−0.20	0.0008	10	−0.16	0.0092	10	0.01	0.9401
11	0.13	0.0228	11	0.05	0.3700	11	0.20	0.0004	11	−0.15	0.0082	11	−0.15	0.0076	11	0.16	0.1371
12	0.13	0.0236	12	0.02	0.7604	12	0.20	0.0006	12	−0.09	0.1024	12	−0.15	0.0120	12	−0.15	0.1319
13	0.13	0.0254	13	−0.01	0.8258	13	0.19	0.0010	13	−0.01	0.7968	13	−0.15	0.0124	13	−0.11	0.3280
14	0.11	0.0548	14	−0.01	0.8540	14	0.19	0.0016	14	0.03	0.6050	14	−0.14	0.0194	14	−0.15	0.1696
15	0.11	0.0618	15	−0.01	0.9078	15	0.19	0.0014	15	0.07	0.2406	15	−0.13	0.0294	15	−0.05	0.6535
16	0.10	0.0784	16	0.03	0.6704	16	0.18	0.0020	16	0.06	0.2742	16	−0.12	0.0370	16	0.00	0.9754
17	0.10	0.0670	17	0.00	0.9690	17	0.18	0.0028	17	0.04	0.4590	17	−0.12	0.0346	17	−0.17	0.1131
18	0.11	0.0428	18	−0.02	0.7438	18	0.17	0.0048	18	0.00	0.9580	18	−0.12	0.0396	18	−0.07	0.5217
19	0.12	0.0370	19	0.01	0.8076	19	0.17	0.0028	19	−0.05	0.4234	19	−0.12	0.0392	19	0.08	0.4360
20	0.12	0.0376	20	0.05	0.4334	20	0.17	0.0056	20	−0.07	0.1826	20	−0.12	0.0392	20	−0.04	0.7054
21	0.11	0.0612	21	0.08	0.1786	21	0.18	0.0038	21	−0.11	0.0548	21	−0.12	0.0478	21	−0.01	0.9278

© 2017 by the authors. Submitted for possible open access publication under the terms and conditions of the Creative Commons Attribution (CC BY) license (http://creativecommons.org/licenses/by/4.0/).
